# Hydrophilic Chitosan Derivatives: Synthesis and Applications

**DOI:** 10.1002/chem.202202156

**Published:** 2022-10-06

**Authors:** Erez Cohen, Elena Poverenov

**Affiliations:** ^1^ Agro-Nanotechnology and Advanced Materials Center Institute of Postharvest and Food Sciences Agriculture Research Organization The Volcani Institute 68 HaMacabim Road Rishon LeZion 7505101 Israel; ^2^ Institute of Biochemistry, Food science and Nutrition Faculty of Agriculture, Food and Environment The Hebrew University of Jerusalem 229 Herzl Street Rehovot 7610001 Israel

**Keywords:** antibacterial, biopolymers, chitosan, hydrophilic substitution, nanoparticles

## Abstract

Polymer alternatives sourced from nature have attracted increasing attention for applications in medicine, cosmetics, agriculture, food, water purification, and more. Among them, chitosan is the most versatile due to its full biodegradability, exceptional biocompatibility, multipurpose bioactivity, and low toxicity. Although remarkable progress has been made in its synthetic modification by using C3/C6 secondary/primary hydroxy (−OH) and the C2 amino (−NH_2_) active sites, its solubility under physiological conditions remains limited and has hampered larger‐scale adoption. This review summarizes different synthetic methods that increase chitosan‘s hydrophilicity and water solubility by using covalent modifications, namely amino acid addition, quaternary ammonium formation, phosphorylation, and carboxymethylation. We also review several applications for each type of substitution in fields such as cosmetics, medicine, agriculture, and water purification, and provide an outlook and perspective for future modifications and implementations.

## Graphical abstract

**A benign and low‐cost alternative** for fossil‐fuel‐sourced polymers, chitosan has tremendous potential because of its bioactivity, biocompatibility, biodegradability, and low toxicity. Synthetic pathways for chitosan derivatives with improved water solubility, and their associated applications, are explored. With the change in global direction towards a lower carbon economy and reduced environmental impact, fine‐tuning chitosan properties is essential for a better future.

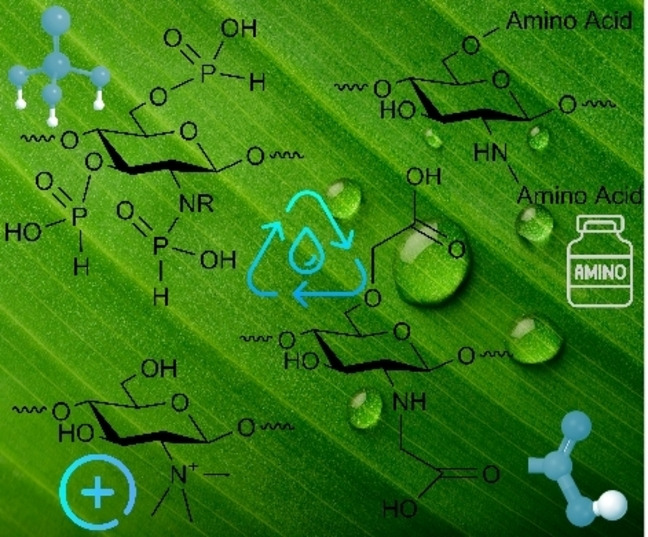

## Introduction

Chitosan, the deacetylated version of the second most abundant biopolymer chitin, has emerged as a promising nature‐sourced functional material.[^1^, ^2^, ^3^] This polysaccharide is produced from renewable resources such as shellfish, exoskeletons of insects, and fungi cell walls^[4]^ and is, therefore, a nontoxic, biocompatible, and biodegradable alternative for various applications.[^4^, ^5^] Its versatile antimicrobial, antifungal, analgesic, and antitumor functionalities enable its utilization in agrotech, foodtech, cosmetics, pharmaceuticals, water purification, textile treatment, and more.[^5^, ^6^, ^7^, ^8^, ^9^, ^10^, ^11^, ^12^, ^13^, ^14^]

Chitosan is an aminoglucopyran copolymer composed of *N*‐acetylglucosamine (GlcNAc) and glucosamine (GlcN) residues with various degrees of deacetylation (DD), commonly ranging from 40–90 % DD.[^5^, ^15^, ^16^] DD is considered a crucial parameter to control its solubility, crystallinity, chelation capability, antimicrobial activity, biodegradability etc.^[4]^ Chitosan has three main reactive sites for chemical modifications: the C3/C6 secondary/primary hydroxy (−OH) and the C2 amino (−NH_2_) groups.[^1^, ^2^, ^5^] These functional groups allow modifications on the polymer backbone to fine‐tune its properties according to desired applications and the design of various products.^[17]^ Recent progress in the field of chitosan chemistry and corresponding applications have been discussed in several reviews and articles.[^1^, ^18^, ^19^, ^20^, ^21^, ^22^, ^23^] However, these reports focus on hydrophobic substitution to induce self‐assembly and aggregation capabilities. This Review is focused on the research concerned with increasing hydrophilicity and water solubility of chitosan. The readers interested in hydrophilic modifications of chitosan will find in this work a summary of recent (2013–2022) developments in the field, major synthetic pathways, their resultant features, and potential applications.

## Synthesis

Chitosan is the only known natural polycation with higher DD% which enhances its charge density potential. As chitosan polymers’ solubility is limited to acidic aqueous solutions, their use in pH‐sensitive applications is rare. Therefore, increasing its solubility under neutral physiological conditions is highly desired.[^14^, ^24^, ^25^, ^26^]

There are several synthetic routes for chitosan‘s hydrophilic modification, whereas in here we will focus on the following: amino acid addition, quaternary ammonium formation, phosphorylation and carboxymethylation.

### Amino acid modification

**Scheme 1:**
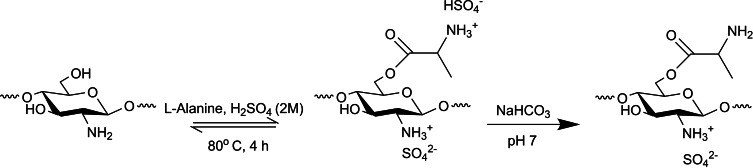
Esterification reaction of chitosan with l‐alanine.^[27]^

Fisher esterification is the central approach for attaching carboxylic acids with alcohol functional groups. Hefni et al. reacted chitosan‘s C6 secondary hydroxy (−OH) with l‐alanine under strongly acidic conditions using dropwise addition of sulfuric acid at room temperature (Scheme 1). After stirring for several hours at 80 °C, the mixture was cooled and adjusted to pH 7 with sodium bicarbonate, followed by several purification steps.^[27]^

The acid acts as esterification catalyst and protects the C2 amine (−NH_2_) from forming salt with the carboxylic acid group. Another option is to use a stronger *para*‐toluene sulfonic acid instead, potentially increasing the reaction yield and later neutralizing and capturing using basic beads such as amberlite.

**Scheme 2:**
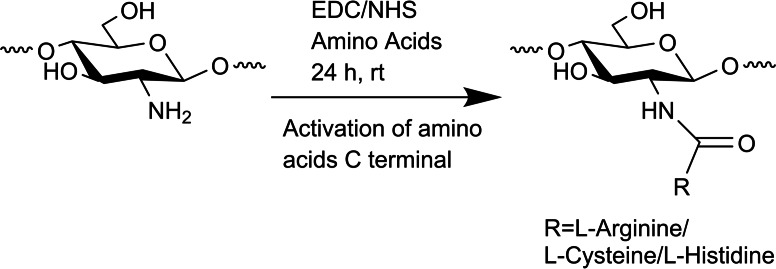
Selective amidation of chitosan with l‐arginine/l‐cysteine/l‐histidine.^[28]^

A different route for amino acid functionalization is the use of C2 amine as the connection point. Gaspar et al. performed selective amidation using 1‐ethyl‐3‐(3‐dimethylaminoisopropyl) carbodiimide (EDC)/*N*‐hydroxysuccinimide (NHS) coupling chemistry by activating the l‐arginine/l‐cysteine/l‐histidine amino acids carboxylate group (Scheme 2).^[28]^

**Scheme 3:**
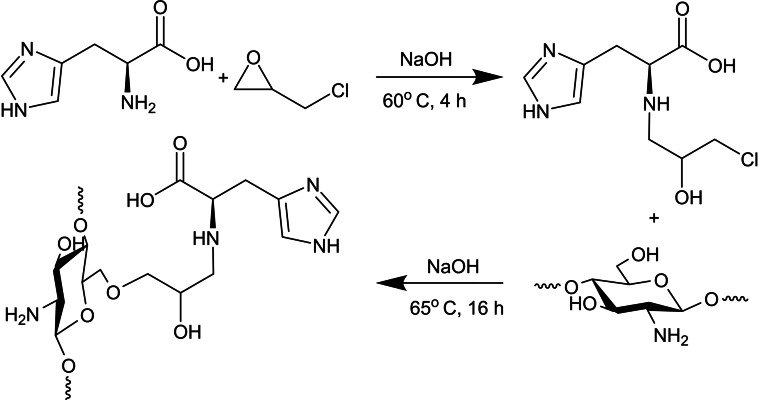
l‐Histidine epichlorohydrin activation followed by chitosan etherification.^[29]^

Beppu and co‐workers employed epichlorohydrin (EPI) epoxide ring‐opening under basic conditions with L‐Histidine amine group; later coupled with chitosan‘s C6 secondary hydroxy for etherification (Scheme 3).^[29]^

### Quaternary ammonium modification

**Scheme 4:**
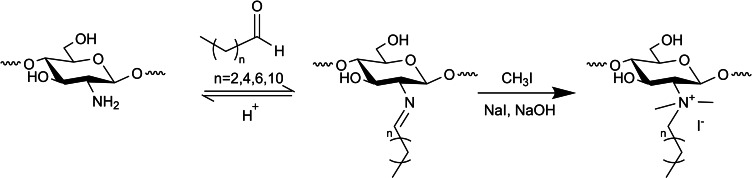
Preparation of quaternary ammonium chitosan polymers by using CH_3_I.^[30]^

Cohen et al. quaternary ammonium formation involved a one‐pot Schiff base reaction to yield the desired imine, followed by product dispersion in *N*‐methyl‐2‐pyrrolidone (NMP) for 12 h at room temperature. NaOH, CH_3_I (excess compared to chitosan's amine), and NaI were added to each dispersion and the reaction continued at 50 °C for 12 h. The final product was collected by precipitation with acetone and dried to obtain the quaternary N‐alkyl chitosan derivatives (Scheme 4).^[30]^

**Scheme 5:**
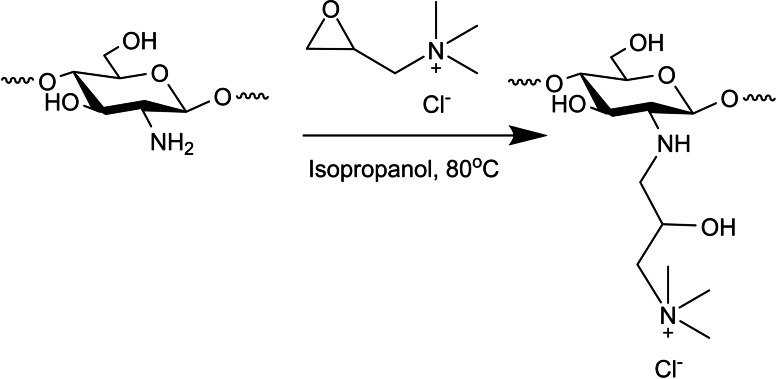
Synthesis of quaternary ammonium chitosan polymers by using GTA.^[31]^

Wen and co‐workers approached this process differently‐instead of forming the quaternary ammonium in situ, they used a pre‐existing one. This was done by grafting 2,3‐epoxypropyl trimethylammonium chloride (GTA) onto chitosan‘s amino groups. Chitosan was dissolved in isopropanol at 80 °C (6 h, pH∼8–9), followed by the addition of GTA aqueous solution, and the reaction reached completion after 24 h at 80 °C. Here, the workup is more tedious than the previous route as it requires two days of dialysis to remove the unreacted GTA and several days of vacuum freeze‐drying (Scheme 5).^[31]^

**Scheme 6:**
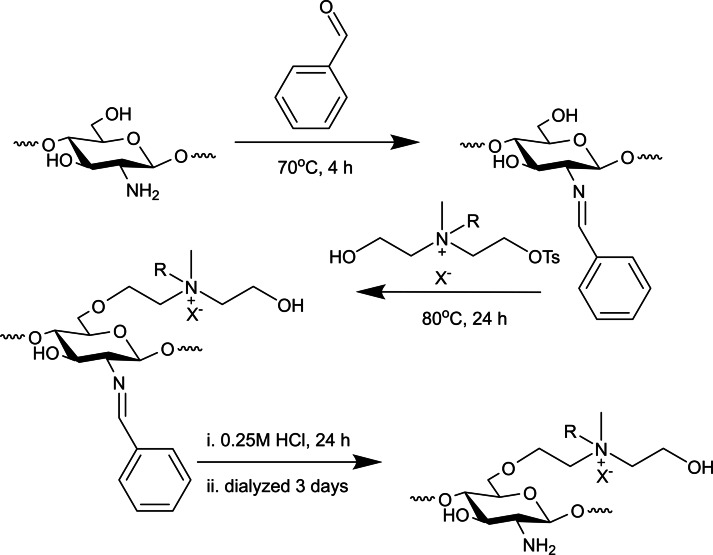
Synthesis of quaternary ammonium on chitosan‘s C6−OH.^[32]^

Wang et al. suggested a different pathway using chitosan‘s reactive C6−OH position, which is very rare in case of quaternary ammonium salts (QAS). C2−NH_2_ position is first protected in the form of imine using benzaldehyde, and later several QAS p‐toluenesulfonate derivatives reacted with the C6−OH position. C2−NH_2_ deprotection with HCl resulted in a precipitate, followed by another precipitation in acetone and finally dissolved in distilled water and dialyzed against distilled water for 3 days. The product is furhter concentrated by precipitating in acetone, and dried under vacuum at 80 °C (Scheme 6).^[32]^

### Phosphorylation modification

**Scheme 7:**
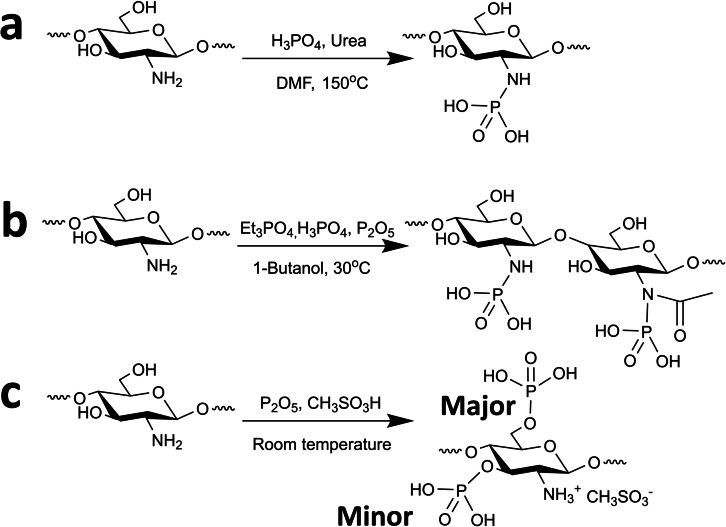
Synthetic routes for chitosan phosphorylation.^[33]^

Wang et al. summarized several common methods for introducing phosphate groups onto chitosan, and provided a systematic comparison of their chemical structure (Scheme 7).^[33]^ The first route involved chitosan‘s reaction with urea and phosphoric acid in DMF at 150 °C for 1 h. The product was filtered, washed, and vacuum dried (Scheme 7a). The second route is the reaction of chitosan with phosphoric acid, Et_3_PO_4,_ followed by gradual addition of P_2_O_5_ under an inert (N_2_) atmosphere. The precipitate was filtered, rinsed with anhydrous ethanol, water, and vacuum dried (Scheme 7b). The last route included the dissolution of chitosan in methanesulfonic acid, the gradual addition of P_2_O_5_, and quenching into ether after completion. The resulting precipitate was filtered, rinsed with ether/acetone, and suspended in distilled water. Finally, the crude was dialyzed, acetone precipitated, and centrifuged to yield the product (Scheme 7c).

**Scheme 8:**
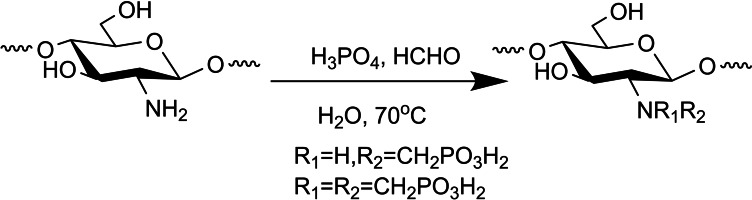
Chitosan phosphorylation with phosphoric acid and formaldehyde.^[34]^

Another route reviewed by Abou Kana and co‐workers described an N‐methylene phosphonic chitosan derivative obtained by phosphoric acid and formaldehyde reaction in water at 70 °C. The crude was dialyzed against distilled water for 2 days, and the final product was attained by freeze‐drying (Scheme 8).^[34]^

**Scheme 9:**
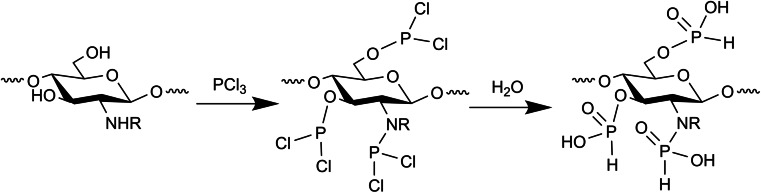
Exhaustive phosphorylation of chitosan by using gas‐phase PCl_3_.^[35]^

Kudzin et al. demonstrated a new method for exhaustive gas‐phase phosphorylation with phosphorous trichloride (PCl_3_), followed by hydrolysis to obtain the final product (Scheme 9).^[35]^

### Carboxymethyl modification

Shariatinia^[36]^ described that carboxymethylation of chitosan at C6−OH and/or C2−NH_2_ positions could result in several carboxymethyl chitosan (CMC) derivatives with enhanced water solubility: O−CMC, N,O−CMC, and N,N−CMC. Under mild alkaline conditions of pH 8–8.5, only the amine group will be activated, whereas, at high alkali concentrations (>25 % NaOH), substitution can occur at C6/C3−OH and C2−NH_2_.^[37]^

**Scheme 10:**
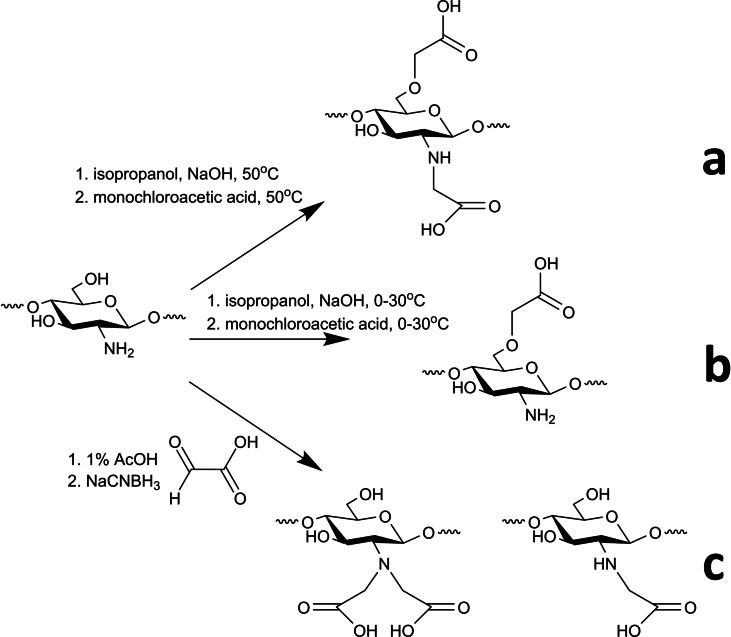
Chitosan carboxymethylation derivatives.[^36^, ^37^, ^38^, ^39^, ^40^]

Doshi et al.[^38^, ^39^] prepared O−CMC by reacting chitosan with 10 % NaOH in isopropanol at 50 °C to form the C6−O anion. Then dropwise addition of monochloroacetic acid yielded the desired product, followed by wash with ethanol, centrifuged, and dried overnight at room temperature. Under these conditions, a mixture of O−CMC and N,O−CMC is usually formed (Scheme 10a), but one can selectively form O−CMC by performing the reaction in a cold bath or at room temperature (Scheme 10b).^[40]^

A mixture of N−CMC and N,N−CMC could be formed exclusively by Schiff base mechanism. Reacting the C2−NH_2_ with an aldehyde in 1 % AcOH, and later reducing it with NaBH_4_ or NaCNBH_3_ avoids the O‐substitution, while N−CMC/N,N−CMC ratio is controlled by reaction conditions (Scheme 10c).^[37]^

## Features and Applications

Chitosan and its modifications have a myriad of technology relevant properties, including antimicrobial,[^32^, ^41^, ^42^] antifungal,[^2^, ^5^, ^43^] wound healing,[^7^, ^44^] antioxidant,[^41^, ^45^] materials chelator[^10^, ^27^, ^38^] and more. In the next paragraphs, we will present various modification‐dependent chitosan applications.

### Amino acid modification

**Figure 1:**
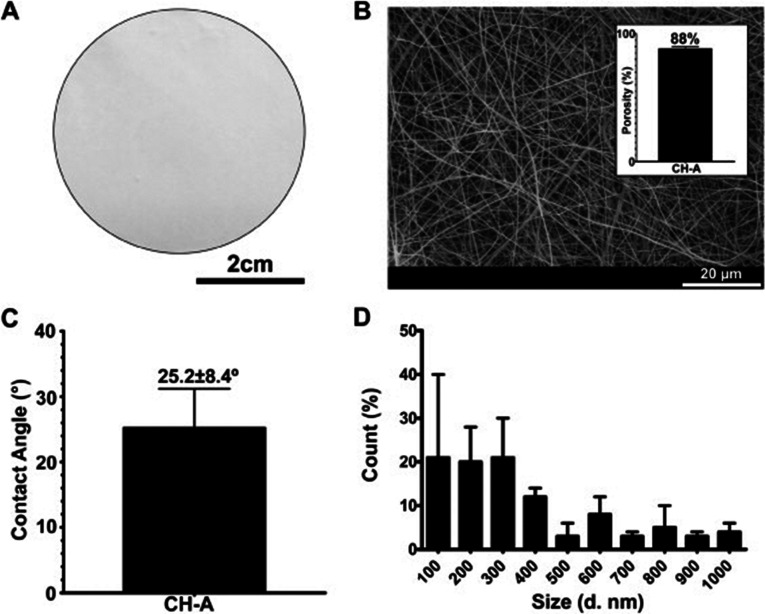
Chitosan‐arginine membrane characterization. A) Macroscopic image of the produced membrane. Analysis of B) membrane porosity and C) membrane surface contact angle. D) Membrane fiber diameter distribution. Reprinted with permission from ref. [46]. Copyright: 2015, Elsevier.

In recent years, chitosan functionalized with amino acids has found a promising use for biomedical purposes, for example, wound healing aimed at re‐establishing the skin‘s native properties.^[7]^ Antunes et al. produced an electrospun membrane from arginine modified chitosan with highly hydrophilic 3D network of fibers, an analog of the human extracellular matrix (Figure 1). The membrane adhered and grew with the human skin, confirming its biocompatibility. The material improved tissue regeneration and promoted faster‐wound closure, supporting its suitability for wound dressing.^[46]^

**Scheme 11:**
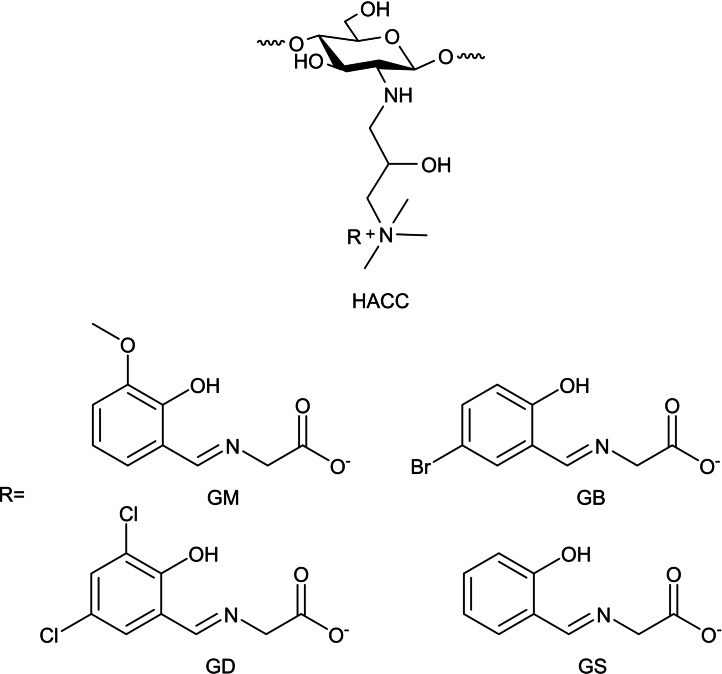
Schiff base derivatives of HACC amino acids.^[41]^

Guo and co‐workers prepared a series of *N*‐2‐hydroxypropyltrimethyl ammonium chitosan products (HACC) bearing amino acid Schiff bases using ion‐exchange reaction (HACGM, HACGB, HACGD, HACGS). The resulting materials are biologically potent with significant improvement in their antibacterial, antifungal, and radical scavenging ability compared to HACC. Specifically, HACGM and HACGB presented substantial inhibitory effects on bacteria and fungi, including *Staphylococcus aureus*, *Escherichia coli*, *Botrytis cinerea*, and *Fusarium oxysporum* f. sp. *cubense*. HACGB inhibition rate on *S. aureus* and *E. coli* could reach 100 % at 0.1 mg mL^−1^ concentration. In contrast, HACGM and HACGB inhibition rate on *B. cinerea* and *F. oxysporum* f. sp. *cubense* reaches 100 % at 0.5 mg mL^−1^ concentration (Scheme 11).^[41]^

Hefni et al. showed that increasing the number of chitosan‘s amino groups using l‐Ala esterification on C6−OH leads to an increase in adsorption efficiency. The uptake efficacy of three heavy metals (Mn^2+^, Pb^2+^, Al^3+^) and total organic carbon (TOC) from wastewater was studied at different concentrations, temperatures, and contact times. While heavy metals uptake was similar between pristine chitosan and substituted one (95.3–99.6 %), TOC uptake by chitosan‐l‐Ala achieved 89 mg/g compared with 50 mg/g for pristine chitosan, corroborating the transformation necessity.^[27]^

Tumor targeting nanomaterials is another promising approach for high‐efficiency anti‐tumoral therapeutics. Correia and co‐workers synthesized multifunctional nanoparticles with dual amino acid‐functionalized chitosan combined with folic acid‐PEG. These nanocarriers (126–176 nm) were complexed with DNA, showing hemocompatibility, cancer cell penetration, and 3.7‐fold increase in gene expression. The nanocarriers presented high specificity towards cancer cells compared to normal cells, promoted p53 tumor suppressor delivery, and decreased tumor spheroid volume.^[28]^

**Figure 2:**
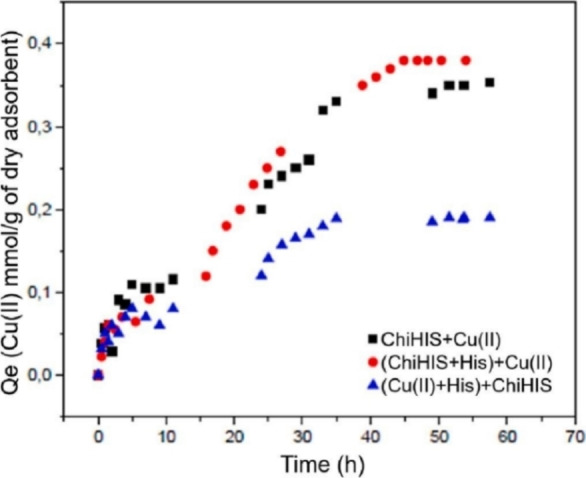
Adsorption kinetics of histidine‐modified chitosan beads at 25 °C, pH 5, and a copper(II) nitrate concentration of 0.47 mmol L^−1^. Reprinted with permission from ref. [29]. Copyright: 2021, Elsevier.

Taketa et al. suggested that chitosan beads modified with histidine can shed light on copper biomolecules interactions, while interfering the bonds between biomolecules and copper ions as a chelator. Chitosan's/histidine in vitro ability for copper ions uptake was tested, increasing copper ions adsorption to 2.47 mmol Cu^II^/gram adsorbent, almost doubled relative to pristine chitosan. Chitosan‐Histidine beads presented different behavior depending on histidine order of addition to the system. Chitosan's/Histidine is a weaker chelator if copper ions‐Histidine bond was already established by first adding histidine to the copper solution, while adsorption decrease wasn't observed in case of chitosan and Histidine simultaneous addition (Figure 2).^[29]^

#### Quaternary ammonium modification

Quaternary ammonium polysaccharides, such as chitosan, have been explored and applied in various antimicrobial applications, including agriculture, food, and medical uses. These materials are very promising due to their availability, low cost, biocompatibility, biodegradability, and specific chemical modifications to tailor their activity.^[47]^ Quaternization of chitosan boosts antibacterial action as a result of amplified electrostatic interaction between the positively charged quaternary ammonium and negatively charged bacterial cell wall.[^14^, ^48^]

**Figure 3:**
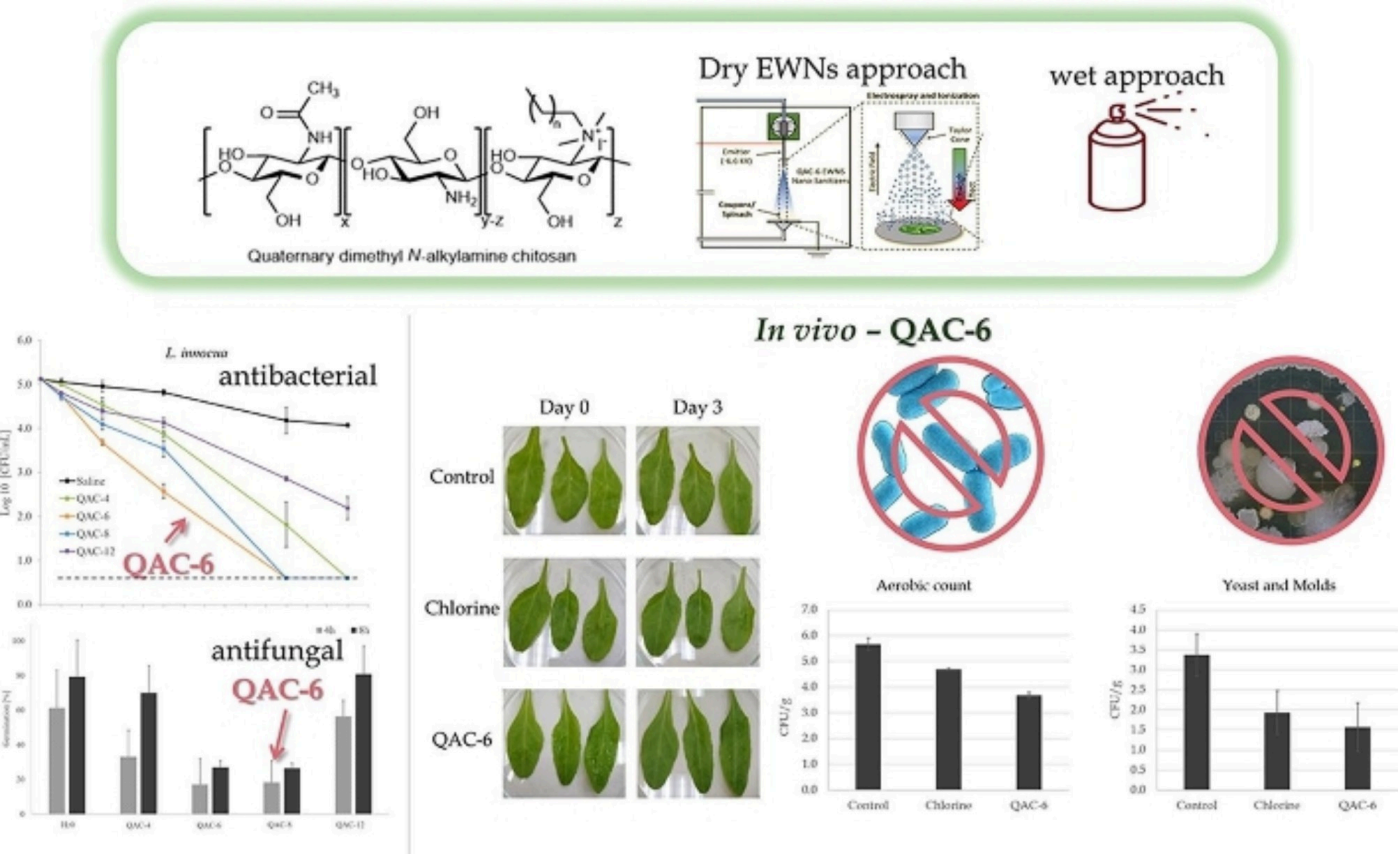
synthesis, application methods, and antibacterial as well as antifungal activity of QAC derivatives. Reprinted with permission from ref. [30]. Copyright: 2022, Elsevier.

Poverenov and co‐workers prepared a series of quaternary dimethyl‐(alkyl)‐ammonium chitosan (QAC), self‐assembling into nanoaggregates. The aggregates were potent against Gram‐positive (*Listeria innocua*) and Gram‐negative (*E. coli*) bacteria, as well as *B. cinerea* fungus. QAC‐6 substituted with hexyl chain exhibited the highest potency, leading to −3.0 and −4.5 log CFU mL^−1^ reduction of *E. coli* and *L. innocua* levels, respectively. The prepared materials were also tested for antimicrobial activity on stainless steel coupons and baby spinach using two methods: wet application (spray) and dry engineered water nanostructure (EWNS) aerosol. Spraying allowed a more significant reduction in bacterial levels, whereas EWNS can reduce bacterial growth using lower concentrations without surface wetting (Figure 3).^[30]^

**Figure 4:**
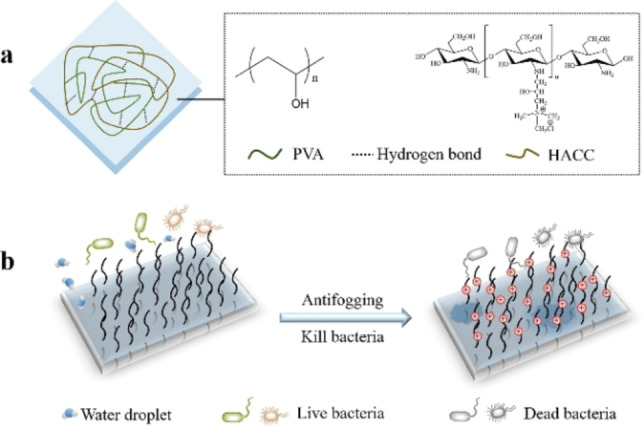
a) Chemical structure of the composite HACC/PVA coatings. b) The dual function of the coating for food packaging. Reprinted with permission from ref. [31]. Copyright: 2020, Elsevier.

Min et al. investigated applications for antifogging and antibacterial food packaging fabricated from quaternary ammonium chitosan (HACC) and polyvinyl alcohol (PVA). Simple solution casting allowed simultaneous coating of the composite components with excellent antifogging (98 % transmittance) and antibacterial properties (up to 99 % bacteria kill) due to its higher water absorption. Its high potency was demonstrated for strawberry packaging, better prolonging their shelf life than single‐use plastics such as polyethylene and polypropylene films (Figure 4).^[31]^

**Figure 5:**
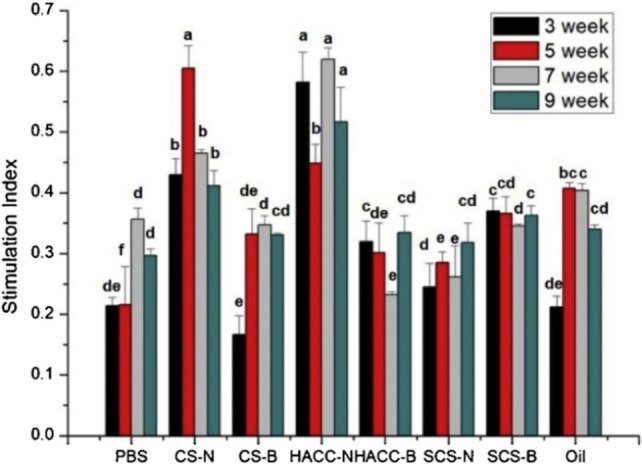
The stimulation index of lymphocyte proliferation in immunized chickens. Eight groups of chickens were immunized with (left to right) PBS, NDV‐loaded chitosan nanoparticle (CS−N), blank chitosan nanoparticle (CS−B), NDV‐loaded hydroxypropyltrimethyl ammonium chloride chitosan/chitosan nanoparticle (HACC−N), blank hydroxypropyltrimethyl ammonium chloride chitosan/chitosan nanoparticle (HACC−B), NDV‐loaded sulfated chitosan nanoparticle (SCS−N), blank sulfated chitosan nanoparticle (SCS−B), and commercial inactivated oil emulsion ND vaccine (Oil). Bars at the same week with the same superscript means no significant difference (*P*<0.05). Reprinted with permission from ref. [50]. Copyright: 2020, Elsevier.

The increased positive charge on quaternized derivatives facilitates cell epithelia crossing and improves mucoadhesive character.^[49]^ As a result, Li and co‐workers used HACC and sulfated chitosan (SCS) as adjuvants of inactivated Newcastle disease (ND) vaccine. Control and encapsulated NDV nanoparticles were prepared with chitosan, HACC/chitosan, and SCS. Chickens were immunized with the commercial oil emulsion vaccine or the prepared nanoparticles, and their humoral immunity levels were evaluated before infecting them with a highly virulent virus. Humoral immunity levels of encapsulated NDV in chitosan and HACC/chitosan were lower than in commercial vaccine, but showed improvement in their cellular immunity levels. Interestingly, encapsulated NDV in chitosan and HACC/chitosan demonstrated comparable prevention effects to the commercial vaccine (Figure 5).^[50]^

**Scheme 12:**
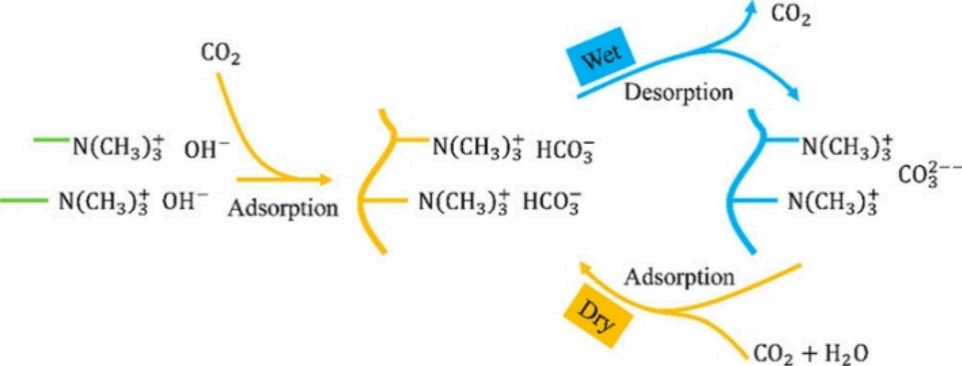
Schematic illustration of reversible CO_2_ capture by humidity swing. Reprinted with permission from ref. [53]. Copyright: 2018, American Chemical Society.

Global warming and greenhouse gas emissions, especially CO_2_, are major concerns for climate stability across the world.^[51]^ Therefore, CO_2_ capture sequestration (CCS) became one of the leading technologies to control CO_2_ emissions from stationary sources, such as power plants. Today's adsorbents are usually solid amine sorbents using temperature swing, for example, amine‐modified SiO_2_ aerogel.^[52]^ Although these materials have high affinity and efficiency for CO_2_ capture, they suffer from high temperatures requirement, leading to high energy consumption and cost. Song et al. developed quaternized chitosan (QCS)/poly(vinyl alcohol) (PVA) hybrid aerogels enriched with amine moieties as a low‐cost material for humidity swing CO_2_ adsorption. The quaternary ammonium sites are the loading area for carbonate or bicarbonate ions. This aerogel CO_2_ capture capacity reached 0.18 mmol g^−1^ at room temperature, 38 % higher than commercial Excellion membrane, providing a large scale and low‐cost alternative for CO_2_ capture using nature‐sourced material (Scheme 12).^[53]^

#### Phosphorylation modification

Phosphorylated chitosan is well known for its high water solubility and metal chelating capability, with tissue regeneration, flame retardancy, and drug delivery applications.^[34]^

**Scheme 13:**
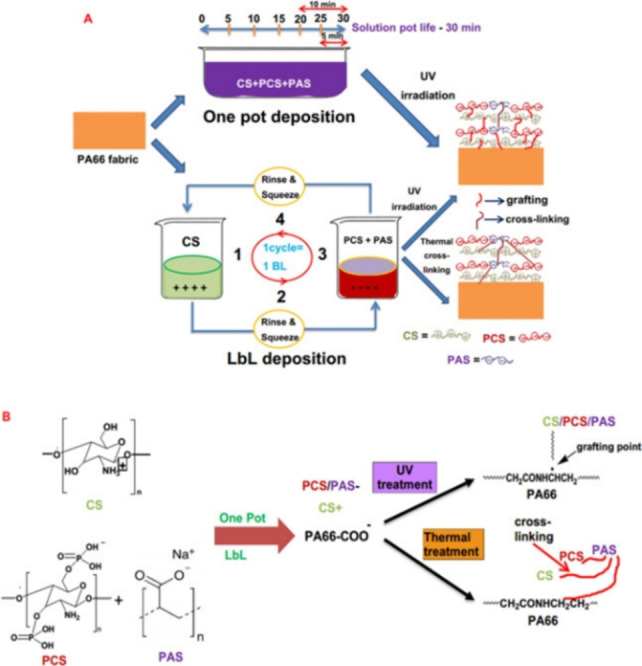
Schematic representation of A) the one‐pot and LbL deposition of PA66 fabrics and B) the mechanism of reaction. Reprinted with permission from ref. [54]. Copyright: 2020, Elsevier.

Hu and co‐workers utilized chitosan, phosphorylated chitosan (PCS) and polyacrylated sodium (PAS) as flame retardant coatings for polyamide 66 textile, using one‐pot and layer‐by‐layer (LbL) deposition methods. Electron microscopy revealed that LbL deposition improves homogeneity compared with one pot, affecting their flame retardancy and thermal properties. Several LbL coated fabrics also involved UV grafting or thermal crosslinking, improving its limiting oxygen index (LOI) by 23 %, while reducing its peak heat release rate (pHRR) by 25 %. In general, LbL was found to be more effective than the one‐pot method, but higher weight gain % results in superior flame retardancy in both cases (Scheme 13).^[54]^

**Figure 6:**
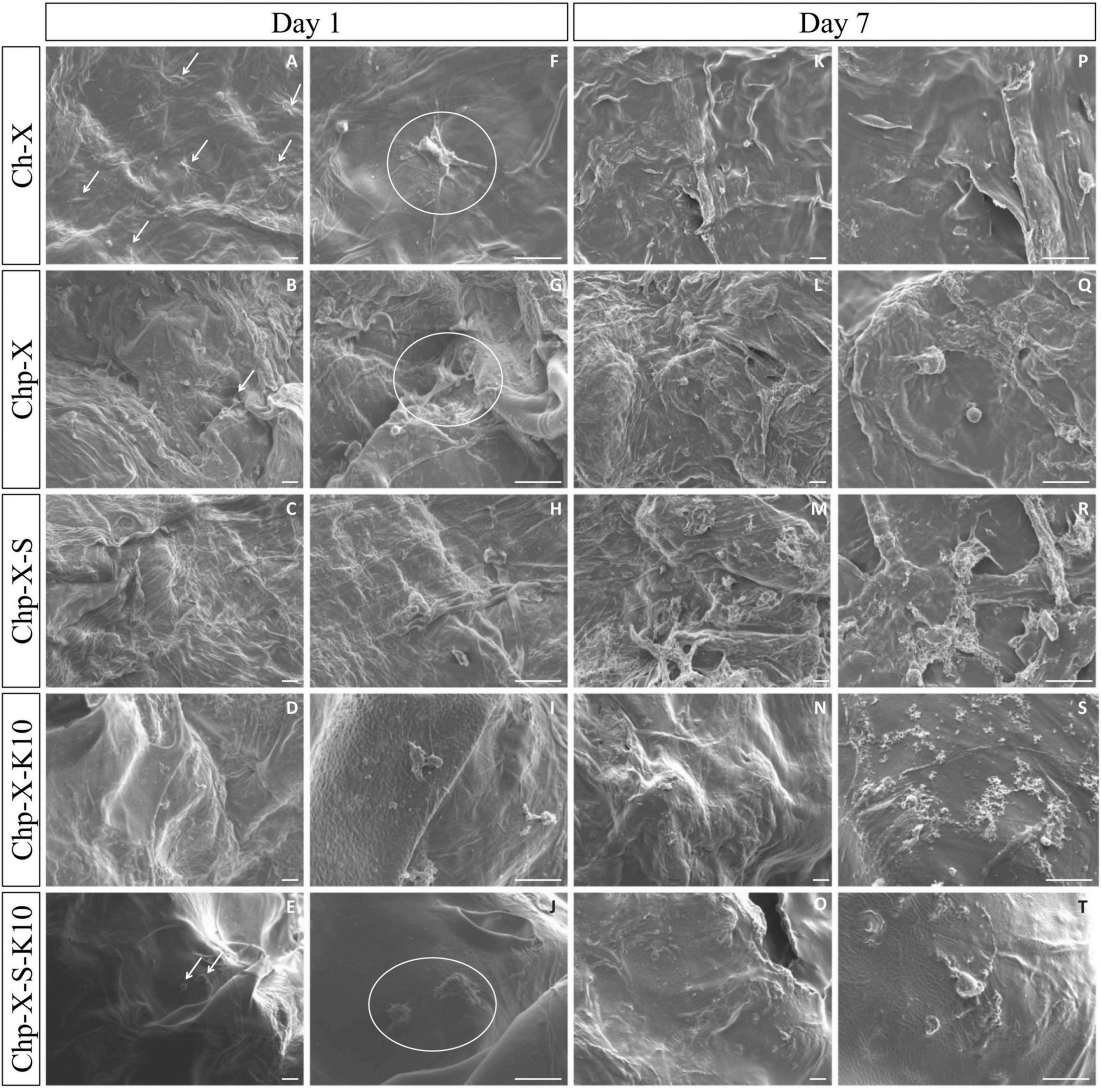
SEM images of cells on the surface of Ch−X (control) and Chp‐X scaffolds prepared in the presence or not of the additives Silpuran® 2130 A/B (S) and Kolliphor® P188 (K) after 1 or 7 days of ADSC culture. White arrows or circles indicate spread cells on the material's surface. Scale bars: 50 μm. Reprinted with permission from ref. [55]. Copyright: 2020, Elsevier.

Another challenge is related to tissue and bone engineering. The periosteum membrane connective tissue covers most of the bone‘s surface, contributing critical cellular and biological elements for fracture and bone healing. Bombaldi de Souza et al. introduced phosphorylated chitosan‐xanthan scaffold functioning as a periosteum substitute to accelerate bone regeneration. The biopolymers composite (with additives) resulted in a reinforced porous matrix with large pore sizes (850–1097 μm), micro‐roughness, and thickness (0.7–3.5 mm) combined with negligible cytotoxicity, thus enabling their usage as periosteum surrogates. Due to their high cytochrome *c* adsorption, concentration of native bone morphogenetic proteins (BMPs) and osteogenesis is expected in vivo (Figure 6).^[55]^

**Scheme 14:**
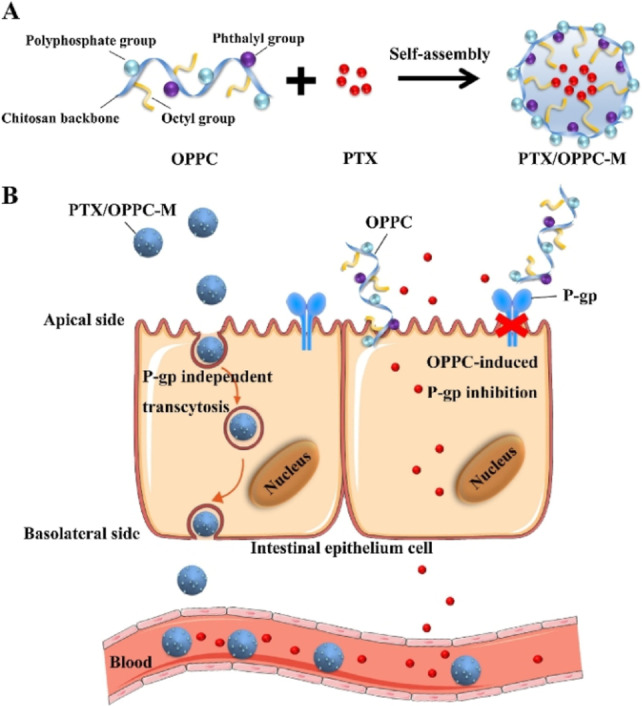
The effect of OPPC micelles in improving the oral absorption of PTX. A) The structure of OPPC and the strategy for constructing the PTX‐loaded OPPC micelles. B) The hypothetical mechanism of improved oral absorption of PTX by OPPC micelles. Reprinted with permission from ref. [56]. Copyright: 2019, Elsevier.

Currently, phosphorylated chitosan derivatives used for drug delivery still require some degree of hydrophobic modification to facilitate lipophilic barriers crossing. The favored route for drug delivery is oral administration due to its accessibility and patient compliance. Zhang and co‐workers developed *N*‐octyl‐*N’*‐phthalyl‐*O*‐phosphoryl chitosan (OPPC) as a carrier for paclitaxel (PTX), a chemotherapy agent. OPPC self‐assembled into micelles and encapsulated PTX with high loading capability. The PTX/OPPC enhanced the drug‘s epithelial permeability and oral bioavailability as detected by in‐situ perfusion and pharmacokinetic studies. These performances are superior to the commonly used taxol in vitro and in vivo, providing a valuable solution for oral bioavailability enhancement (Scheme 14).^[56]^

#### Carboxymethyl modification

**Scheme 15:**
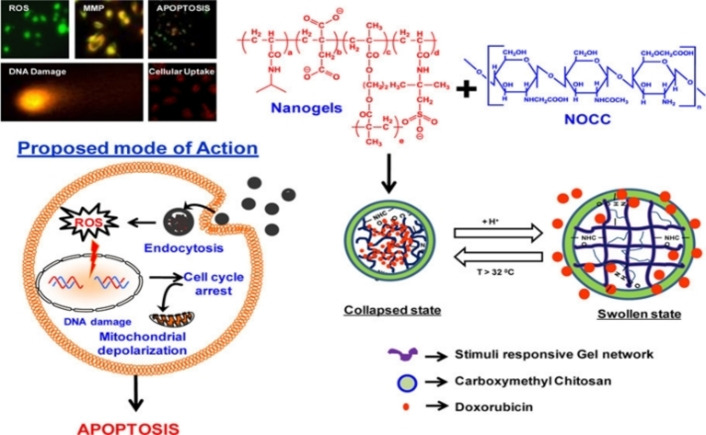
Schematic representation of nanogel components, swelling/shrinking cycle, and proposed mode of action for encapsulated DOX. Reprinted with permission from ref. [57]. Copyright: 2016, American Chemical Society.

Chitosan's poor solubility in water under physiological conditions could be overcome by its water‐soluble carboxymethyl modified versions. Drug delivery, cosmetics, bioimaging, and tissue engineering are among their typical applications. Carboxymethyl chitosan can potentially deliver antibacterial, antifungal, and anticancer drugs, as well as genetic material and proteins.^[36]^ Verma et al. prepared a series of temperature/pH responsive crosslinked nanogels from poly(NIPAAm‐IA‐AMPS) and ethylene glycol dimethacrylate (EGDMA). The nanogels responsiveness was tuned by controlling the copolymerization components ratio. The nanogels were coupled with N,O‐carboxymethyl chitosan (NOCC) to induce swelling at low pH, controlling their nanoscale dimensions by cycling between high temperature (shrinkage) and acidic pH (swelling). Using physical entrapment, an anti‐cancer drug named Doxorubicin (DOX) was encapsulated in the nanogels, providing a controlled release profile under physiological conditions. Moreover, the nanogels provide specific cytotoxicity toward cancer cells, emphasizing their improved therapeutic efficacy and potentially reduced side effects (Scheme 15).^[57]^

CMC‐based products are very attractive in cosmetics because of their high water solubility and similarity with extracellular matrix polysaccharides, meaning: gelator capabilities, significant hydrodynamic volume, high viscosity, and large osmotic pressure.^[58]^ Upon skin contact, CMC forms a protective and moisturizing elastic film that smoothens and protects the skin, providing better hydration even compared to hyaluronic acid.^[20]^

**Figure 7:**
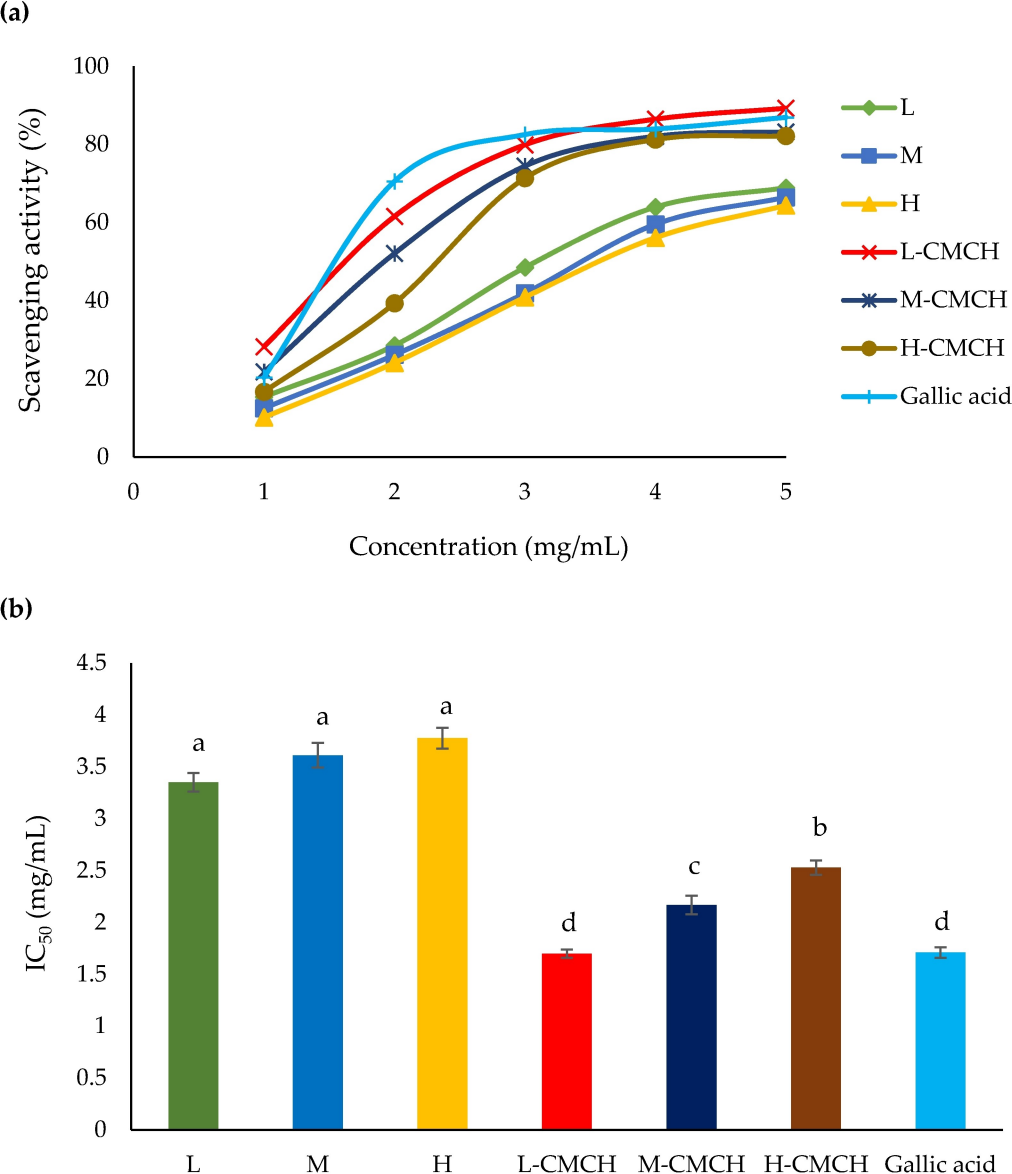
a) DPPH radical scavenging activity [%] and b) IC_50_ of L, M, H, L–CMCH, M–CMCH and H‐CMCH. Different letters (a–d) indicate a significant difference between treatments (*p*≤0.05). Reprinted with permission from ref. [59]. Copyright: 2020, MDPI.

Phimolsiripol and co‐workers investigated carboxymethyl chitosan (CMCH) with different molecular weights regarding their antioxidant and moisturizing characteristics (L–CMCH 50–190 kDa, M–CMCH 210–300 kDa, H‐CMCH 310–375 kDa). A water solubility improvement of 89–96 % was observed compared with chitosan, while higher MW increased viscosity. Testing their antioxidant capabilities (IC_50_) with respect to DPPH and ABTS radical scavenging activity revealed values of 1.70 and 1.37 mg mL^−1^, respectively (L–CMCH). H‐CMCH displayed the highest moisturizing effect than the other MW studied, better than untreated skin, distilled water, propylene glycol, and pristine chitosan alternatives. These findings suggest H‐CMCH is a good emulsion stabilizer and thickening agent, while L–CMCH is a successful antioxidant for cosmetics applications (Figure 7).^[59]^

**Scheme 16:**
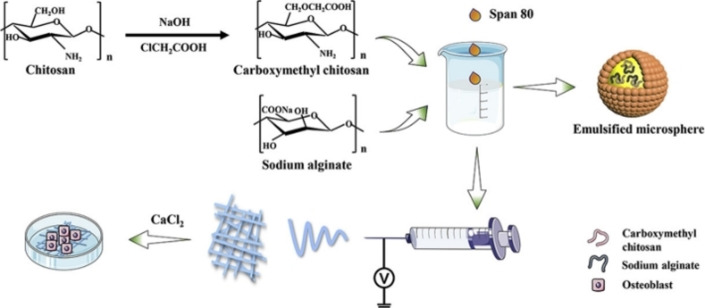
Schematic representation of PCL/CMCS/SA emulsion formation, microfiber generation using electrospinning, and osteoblast adhesion. Reprinted with permission from ref. [60]. Copyright: 2020, Elsevier.

Tissue engineering is another desired application, specifically since CMC scaffolds are biodegradable when the new tissue is regenerated, having minimal toxic degradation products and inflammatory responses.^[36]^ Tao et al. developed a composite of polycaprolactone (PCL)/carboxymethyl chitosan (CMCS)/sodium alginate (SA), serving as a microfibrous periosteum alternative. The periosteum facilitates osteogenesis by providing necessary building blocks; therefore, artificial periosteum, guiding bone tissue regeneration is highly desired. Emulsion electrospinning of PCL/CMCS/SA was used to fabricate the microfibers, later studied for their physicochemical properties, osteoinductive capacity, and biocompatibility to MC3T3‐E1 osteoblasts. PCL/CMCS/SA microfibers had an average diameter of 2.381±1.068 μm, outstanding tensile strength, low cytotoxicity, and satisfying osteoblast adhesion, which is very promising for bone defect regeneration (Scheme 16).^[60]^

**Scheme 17:**
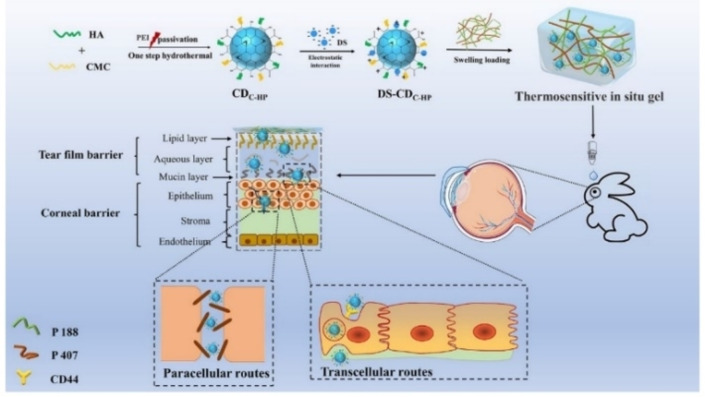
Illustration of the fabrication and possible in‐vivo process and penetration behavior of DS−CD_C−HP_‐Gel. The penetration behavior might be induced by transcellular and paracellular routes. Reprinted with permission from ref. [61]. Copyright: 2021, MDPI.

An additional application of bioimaging, a method of non‐invasive visualization of biological samples, is becoming increasingly important. The technique can rely on various sources, including fluorescence, electrons, and X‐rays for imaging. Pan and co‐workers explored a composite for topical administration of diclofenac sodium (DS), composed of hyaluronic acid and CMC carbon dots CD_C−HP_. The CDs were synthesized by pyrolysis using one‐step hydrothermal method, later embedded in thermoresponsive gel of poloxamer 407 and 188. In vitro studies showed the DS−CD_C−HP_‐Gel supported controlled release for 12 h, whereas, ex vivo fluorescence distribution in ocular tissues proved its bioimaging and tracing capabilities. Furthermore, DS−CD_C−HP_‐Gel reduced tear elimination and increased humor bioavailability. The system was easily administered and can potentially lead to better patient compliance and reduced medicating occurrence (Scheme 17).^[61]^

Wound healing using CMC and rigid rod‐like dialdehyde‐modified cellulose nanocrystal (DACNC) was addressed by Huang et al. Dynamic Schiff‐base crosslinking between CMC amines, and DACNC aldehydes was utilized to create a rapidly self‐healing hydrogel. The DACNC nanoinforcing fillers nature restricted the soft CMC chains motion and improved the hydrogel strength. The hydrogel enables painless removal of dressing by dissolving in amino acid solution, while acting as extracellular matrix for cell growth with cell viability of 97.3 % after one week. Only 0.6 % of the wound area remained after two weeks of healing, as confirmed in vivo, showing promising potential for burn patients treatment.^[62]^

Three‐dimensional printing biofabrication is another innovative technique that can produce personalized on‐demand bioscaffolds carrying cells and other bioactive agents for regenerative treatments. Wang and co‐workers used N,O‐carboxymethyl chitosan (N,O−CMC) 3D template as an extracellular matrix alternative. To do so, the polymer was functionalized using osteogenically active polyphosphate through Ca^2+^ bridges. This complex was found to be robust and fits printing technology, as demonstrated using printed tissues inducing SaOS‐2 bone‐like cells to biomineralization. Its in‐vivo regeneration activity was superior to β‐TCP, and β‐TCP plus silica, currently established materials.^[63]^

Due to its high hydrophilicity, carboxymethyl chitosan was also used for real‐time, noninvasive detection of glucose from tears as an early diagnostic for chronic diseases such as diabetes. Zou et al. combined nitrogen‐doped graphene (highly electroactive) with carboxymethyl chitosan using one‐step ball milling synthesis, avoiding flammable/toxic chemicals and high temperatures. The glucose biosensor was immobilized on the glucose oxidase enzyme, presenting a broad linear range at 12 mM, high sensitivity at 9.7 μA mM^−1^ cm^−2^, and a good detection limit of 9.5 μM. Remarkably, the sensor remained active even after one month of storage and is biocompatible to ophthalmologic cells.^[64]^

## Summary and Outlook

This Review has highlighted and discussed recent developments in the synthesis and potential applications of hydrophilic derivatives of chitosan. The routes reviewed here used chitosan‘s reactive sites, C3/C6 secondary/primary hydroxy (−OH) and the C2 amino (−NH_2_) groups, to undergo four main transformations: amino acid addition, quaternary ammonium formation, phosphorylation, and carboxymethylation. The driving force for introducing these functional groups is to overcome chitosan‘s major drawback: limited applications due to its insolubility in neutral and basic pH media. The newly formed derivatives displayed improved antimicrobial, antitumoral, encapsulation, and materials chelation capabilities, possibly introducing these biocompatible and biodegradable materials into diverse sectors, including cosmetics, pharmaceuticals, textile treatment, water purification, and bioimaging.

A critical challenge is to develop new synthetic pathways toward more stimuli‐responsive versions of chitosan‐based materials. Recent progress towards this goal was achieved by using click‐chemistry principles, specifically thiol‐ene photochemistry. Up to now, most chitosan hydrogels have lost their pH responsiveness due to C2 amino reaction with various functional groups. To overcome that, researchers selectively modified C6−OH with an allyl group, while Schiff base imine protection/deprotection was employed on the C2 amino group. This led to a UV‐crosslinkable gel with PEG‐SH that maintained its pH responsiveness, potentially providing a new drug‐delivery agent.

Carbon quantum dots (CD) from nature‐sourced materials are a promising alternative to conventional and toxic quantum dots (QD) such as CdSe, PbSe, and InGaAs. Such nanoparticles can have applications in light energy conversion, photocatalysis, sensors, and more. A current study reported nitrogen‐doped graphene quantum dots (NGQDs), economically synthesized from the highly abundant chitosan precursor by microplasma chemistry control under ambient conditions. The NGQDs were capable of label‐free, rapid, and ionic, stable, wide range pH sensing from 1.8 to 13.6. The generic approach employed here opens the door to chitosan‐derived nanomaterials that are biocompatible and can be used in vivo and in vitro.

Chitosan, especially in its more water‐soluble versions presented here, possesses a unique set of properties like complete biodegradability, outstanding biocompatibility, versatile bioactivity, and low toxicity. Our vision is that as the world progresses toward a lower carbon footprint and better end‐of‐life solutions against conventional toxic/non‐degradable materials, the demand for eco‐friendly biopolymers within current applications and beyond will continue to grow rapidly.

## Conflict of interest

The authors declare no conflict of interest.

1

## Biographical Information

*Erez Cohen is a postdoctoral fellow at the Volcani Institute, Agricultural Research Organization. He received his B.Sc. in chemistry from Tel‐Aviv University in 2010, and his M.Sc. (2013) and Ph.D. (2018) in chemistry from the Weizmann Institute of Science. He then joined the University of Oxford (UK) Materials Department as Research Manager of the Energy Storage Facility. His current research interests focus on developing robust and adaptive polysaccharides derivatives based on noncovalent interactions for agro and food tech applications*.

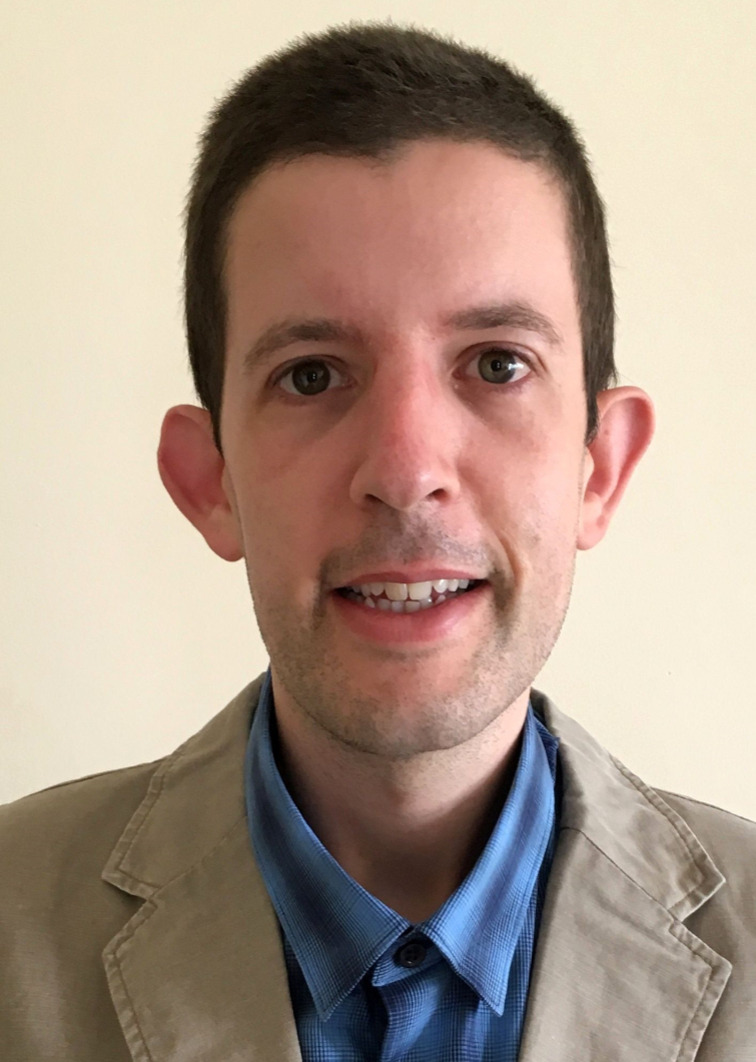

## Biographical Information

*Elena Poverenov is a Rank A researcher (equal to Associate Professor) at the Volcani Institute, Agricultural Research Organization. She received her B.Sc. in chemistry from Bar‐Ilan University in 2001, and her M.Sc. (2004) and Ph.D. (2009) in chemistry from the Weizmann Institute of Science. She then continued her postdoctoral research at the Weizmann Institute of Science. Her research group has distinguished itself for its expertise in developing new materials and advanced delivery systems based on biopolymer raw materials, specifically polysaccharides*.

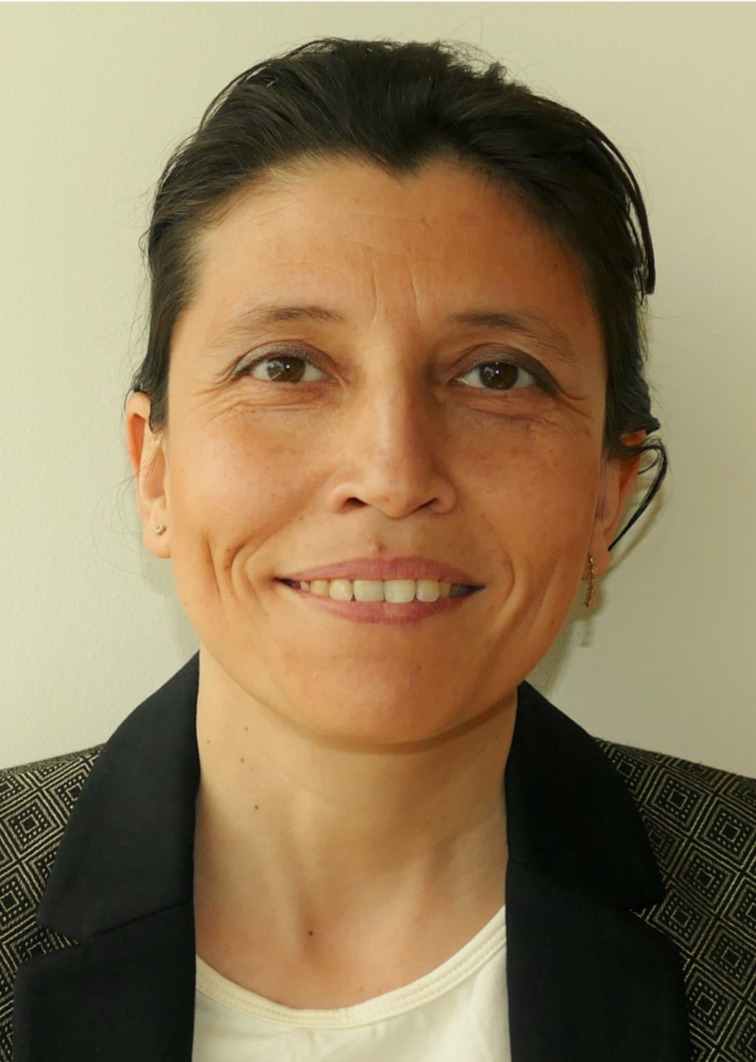

## Data Availability

Data sharing is not applicable to this article as no new data were created or analyzed in this study.
